# Distinct Muscle Biopsy Findings in Genetically Defined Adult-Onset Motor Neuron Disorders

**DOI:** 10.1371/journal.pone.0151376

**Published:** 2016-03-21

**Authors:** Manu Jokela, Sanna Huovinen, Olayinka Raheem, Mikaela Lindfors, Johanna Palmio, Sini Penttilä, Bjarne Udd

**Affiliations:** 1 Division of Clinical Neurosciences, Turku University Hospital and University of Turku, Turku, Finland; 2 Neuromuscular Research Center, Department of Neurology, University Hospital and University of Tampere, Tampere, Finland; 3 Department of Pathology, Fimlab Laboratories, Tampere University Hospital, Tampere, Finland; 4 Department of Neurology, Vasa Central Hospital, Vasa, Finland; 5 Folkhälsan Genetic Institute, Department of Medical Genetics, Helsinki University, Helsinki, Finland; University of Edinburgh, UNITED KINGDOM

## Abstract

The objective of this study was to characterize and compare muscle histopathological findings in 3 different genetic motor neuron disorders. We retrospectively re-assessed muscle biopsy findings in 23 patients with autosomal dominant lower motor neuron disease caused by p.G66V mutation in *CHCHD10 (SMAJ)*, 10 X-linked spinal and bulbar muscular atrophy (SBMA) and 11 autosomal dominant *c9orf72*-mutated amyotrophic lateral sclerosis (c9ALS) patients. Distinct large fiber type grouping consisting of non-atrophic type IIA muscle fibers were 100% specific for the late-onset spinal muscular atrophies (*SMAJ* and *SBMA*) and were never observed in c9ALS. Common, but less specific findings included small groups of highly atrophic rounded type IIA fibers in *SMAJ/SBMA*, whereas in c9ALS, small group atrophies consisting of small-caliber angular fibers involving both fiber types were more characteristic. We also show that in the 2 slowly progressive motor neuron disorders (*SMAJ* and *SBMA*) the initial neurogenic features are often confused with considerable secondary “myopathic” changes at later disease stages, such as rimmed vacuoles, myofibrillar aggregates and numerous fibers reactive for fetal myosin heavy chain (dMyHC) antibodies. Based on our findings, muscle biopsy may be valuable in the diagnostic work-up of suspected motor neuron disorders in order to avoid a false ALS diagnosis in patients without clear findings of upper motor neuron lesions.

## Introduction

Histopathological analysis of muscle is not required for the diagnosis of amyotrophic lateral sclerosis (ALS) and neurologists rarely perform muscle biopsy on motor neuron disease patients when the diagnosis is not in doubt. Nevertheless, several neurogenic entities may clinically simulate myopathies, and some myopathies such as *LMNA-* mutated muscular dystrophies and inclusion body myositis may also show neurogenic features. In these cases muscle biopsy is likely to provide diagnostically useful information. Slow (type I) and fast (type II) muscle fibers are usually distributed in a checkerboard or mosaic-like pattern in healthy muscles. Because lower motor neurons define the fiber types of innervated muscle fibers (either fast or slow), reinnervation of denervated fibers by axonal sprouts from surviving motor neurons typically leads to grouping of muscle fibers of the same type. This is considered the myopathological hallmark of neurogenic change.

We recently described a new slowly progressive type of adult-onset spinal muscular atrophy caused by a c.197G>T p.G66V mutation in *CHCHD10* [1] (SMAJ, OMIM #615048). Many of the patients had initially been diagnosed as ALS, which carries a much less favourable prognosis than SMAJ. Primary diagnostic evaluations in our SMAJ patients indicated that muscle biopsy findings were dissimilar in SMAJ compared with ALS, and therefore a study to detail the differential features was needed. To this end, we compared three distinct genetic motor neuron diseases: spinal and bulbar muscular atrophy (SBMA), c9orf72-related ALS (c9ALS) and SMAJ. In addition, for selected SMAJ cases we evaluated the expression of CHCHD10 protein in muscle tissue by immunohistochemistry, and examined skeletal muscle mitochondrial ultrastructure by electron microscopy.

## Materials and Methods

### Patient characteristics

Clinical features of the SMAJ patients have previously been reported^1^. All patients were genetically confirmed. CAG-repeat numbers in SBMA-patients ranged between 40 and 53 (median 45) repeats. SBMA/SMAJ patients had usually been symptomatic for several years before undergoing first neurological examinations (Table 1). 1 SBMA and 4 SMAJ patients had disease durations of more than 20 years. Common features in SBMA and SMAJ patients were cramping and fasciculations, lower limb onset of weakness and reduced or absent tendon reflexes. 8 c9ALS patients died or were respirator-dependent within a mean of 3.3 years after disease onset (range 2–5.5 years) and 3 were alive but disabled 1.5–3.5 years from onset.

**Table 1:** Comparison of muscle histopathological findings in different genetic motor neuron disorders. All P values in the right-most column apply to comparisons of both C9ALS versus SBMA and C9ALS versus SMAJ. None of the differences between SMAJ and SBMA groups were statistically significant. SMAJ = spinal muscular atrophy, Jokela type, SBMA = spinal and bulbar muscular atrophy, C9ALS = amyotrophic lateral sclerosis caused by pathological hexanucleotide expansion in the gene *C9orf72*. NA = not available.

	Patient group			
	C9ALS (12 biopsies, 11 patients mean age 63, SD 9.7 years)	SBMA (10 biopsies, mean age 59, SD 8.7 years)	SMAJ (24 biopsies, 23 patients, mean age 55, SD 13 years)	
Median diseaseduration at biopsy (range)	1 (0.5–1.5)years	7.5 (2–20) years	4.5 (0–24) years	P value (Chi square test)
Mild or extensive fiber type grouping	67%(8/12)	10(100%)	24 (100%)	
Duration 0–5 years	67%	100%	100%	
Duration >6 years	NA	100%	100%	
Extensive fiber type grouping	25%(3/12)	90%(9/10)	88%(21/24)	<0.05
Duration 0–5 years	25%(3/12)	80% (4/5)	92%(12/13)	
Duration > 6 years	NA	100%(5/5)	82% (9/11)	
Large fiber typegrouping composed of non-atrophic IIA fibers	0% (0/12)	40%(4/10)	58%(14/24)	<0.05
Duration 0–5 years	0%	60% (3/5)	85% (11/13)	
Duration > 6 years	NA	20% (1/5)	27% (3/11)	
Small group atrophy	92%(11/12)	30%(3/10)	33%(8/24)	<0.05
Duration 0–5 years	92%(11/12)	40% (2/5)	38%(5/13)	
Duration > 6 years	NA	20% (1/5)	45%(5/11)	
Large group atrophy	25%(3/12)	50%(5/10)	50%(12/24)	Not significant
Duration 0–5 years	25%(3/12)	60% (3/5)	38%(5/13)	
Duration > 6 years	NA	40% (2/5)	64% (7/11)	
Groups of small rounded IIA/IIX fibers	33% (4/12)	80% (8/10)	79%(19/24)	<0.05
Duration 0–5 years	33% (4/12)	80% (4/5)	77%(10/13)	
Duration > 6 years	NA	80% (4/5)	82% (9/11)	
Small group atrophy with mixed fiber types	75% (9/12)	10% (1/10)	33% (8/24)	<0.05
Duration 0–5 years	75% (9/12)	20% (1/5)	23% (3/13)	
Duration > 6 years	NA	0%(0/5)	45% (5/11)	

In patients with only cramps but no clinical muscle weakness or atrophy, we arbitrarily defined the disease duration as 0 years, if the muscle biopsy had been taken at the same time when EMG confirmed a neurogenic disorder. All biopsies were obtained from a clinically or electrophysiologically affected muscle.

Patients provided a written informed consent for the muscle biopsies. This retrospective study was approved by the Tampere University Hospital (Tampere, Finland) Ethics Committee.

### Muscle biopsies

We re-assessed all muscle biopsies from 23 SMAJ, 10 SBMA and 11 c9ALS patients evaluated at Tampere Neuromuscular Research Center. 1 c9ALS and 1 SMAJ patient underwent 2 biopsies each. The quadriceps muscle, usually vastus lateralis, was biopsied in 7 SMAJ, 6 SBMA, 8 c9ALS patients and tibialis anterior muscle in 12 SMAJ, 4 SBMA and 2 c9ALS patients. Soleus, deltoid and medial gastrocnemius muscles were biopsied in 3 (2 SMAJ, 1 c9ALS), 2 (1 SMAJ and 1 c9ALS) and 2 SMAJ patients, respectively. Fresh frozen cryostat sections of muscle tissue were stained with routine histochemical and immunohistochemical techniques [2,3]. In 1 SBMA patient, only MyHC double staining was performed and in 2 other SBMA patients, oxidative enzyme stainings had not been performed. All other c9ALS, SMAJ and SBMA biopsies were assessed for haematoxylin&eosin (H&E), Gomöri trichrome, major histocompatibility complex (MHC) class I, fetal and neonatal myosin heavy chains (MyHCd and MyHCn), and the oxidative enzyme stainings: nicotineamide adenine dinucleotide tetrazolium reductase (NADH-TR) and combined cytochrome oxidase (COX)/succinate dehydrogenase (SDH).

Additional immunohistochemical stainings were performed with the Ventana BenchMark immunoautomate (Roche Tissue Diagnostics, Inc AZ 85755) in 1 SBMA and 3 SMAJ patients, and the antibodies and their dilutions are listed in Table 2. Ventana peroxidase based detection kit (UltraView Universal DAB detection system kit, Roche Tissue Diagnostics, Inc. AZ 85755) was used for visualization.

**Table 2:** Antibodies used for immunohistochemistry, dilutions and suppliers.

Antibody	Dilution	Supplier
Myotilin	1:50	Leica Biosystems, Novocastra
AlphaBC	1:10	Leica Biosystems, Novocastra
Desmin	1:800	Biogenex
Dystrophin-2	1:100	Leica Biosystems, Novocastra
SMI-31	1:1000	Biosite
TDP-43	1:175	Proteintech Europe
P62 (SQSTM1)	1:100	Santa Cruz Biotechnology
Ubiquitin	1:300	Dako
LAMP2	1:50	Southern Biotech
LC3b	1:50	Cell Signaling Technology
VCP	1:600	Thermo Scientific
FHL-1	1:200	Lifespan Biosciences
Myosin Heavy Chain, Slow	1:200	Leica Biosystems
Myosin Heavy Chain, A4.74 (fast)	1:100	Developmental Studies Hybridoma Bank
Myosin Heavy Chain, developmental	1:40	Leica Biosystems
Myosin Heavy Chain, neonatal	1:20	Leica Biosystems
CHCHD10	1:10	Novus Biologicals

Biopsies of 3 SMAJ patients were used for CHCHD10 immunohistochemistry and ultrastructural studies. Semithin and ultrathin sections were performed with routine methods [4] and the specimens were examined with JEOL 1400 transmission electron microscope (JEOL, Japan). Electron micrographs were obtained with the Olympus-SIS Morada digital camera (Olympus Soft Imaging Solutions, Munster, Germany).

**Fig 1:**
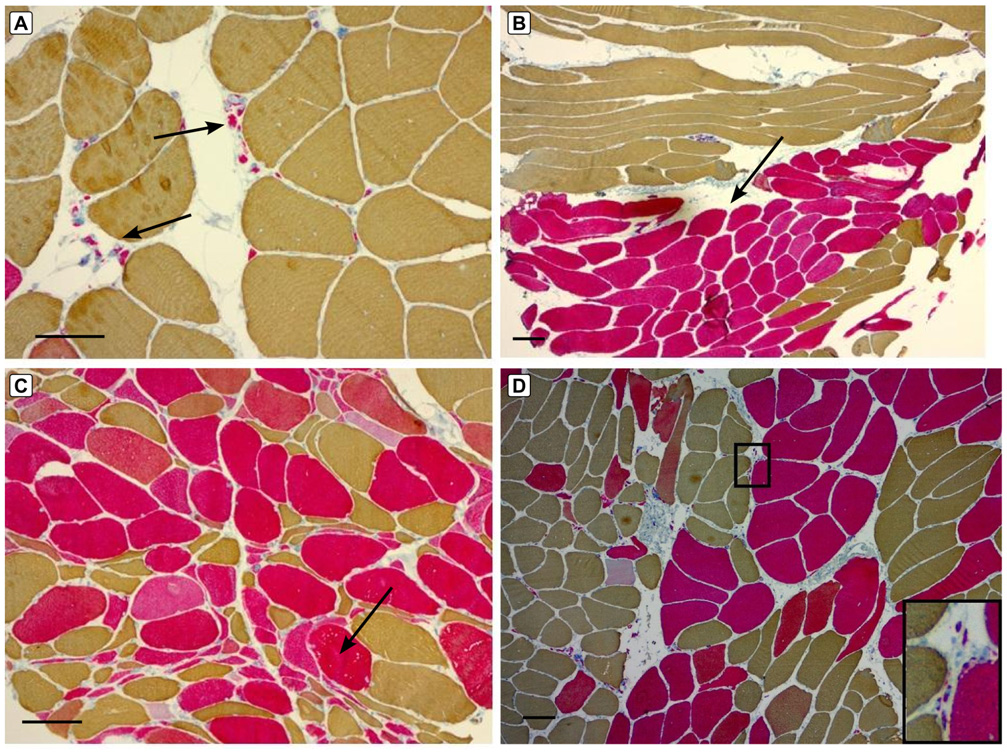
Neurogenic muscle biopsy findings in *SMAJ, SBMA* and *c9ALS*. Small groups of rounded type IIA fibers (arrows) in a vastus lateralis muscle biopsy of a 48-year-old male with SMAJ (A). Extensive fiber type grouping involving non-atrophic type IIA fibers (arrows) shown in a tibialis anterior biopsy of a 55-year-old SMAJ female patient (B). Groups of angulated atrophic fibers with mixed fiber types (arrows) in a vastus lateralis muscle of a 52-year-old female patient with c9ALS (C). Small groups of extremely atrophic type IIA fibers (inset) in a tibialis anterior biopsy of a 60-year-old male patient with SBMA (D). Figs A-D: myosin heavy chain double stain immunohistochemistry showing type IIA fibers in red, IIX fibers in blue and type I fibers in brown color. Scale bar = 100 micrometers

Fiber type grouping was defined as the presence of both type I and type II fibers clustered in solid groups. We subdivided this into “mild grouping” when the groups contained 1 fiber of the same fiber type entirely surrounded by fibers of the same type, and “extensive grouping” when at least 2 fibers of the same type were entirely surrounded by fibers of the same type. Small group atrophies contained two or more adjacent angular fibers of the same fiber type (either type I or II) or consisted of mixed fiber types (both types I and II). Groups of highly atrophic IIA/IIX fibers consisted of several adjacent, mostly rounded, fibers with diameters of <20 micrometers (Fig 1). Large group atrophies were composed of extensive fiber type grouping of atrophic fibers, and non-atrophic IIA fiber groups had to display extensive fiber type grouping of non-atrophic type IIA fibers, ie. with diameters of more than 50–60 micrometers.

### Statistical analysis

Mean and standard deviation or median with range, were used to report descriptive statistics, as appropriate. Comparisons of dichotomous variables between groups were performed with Chi Square test. P = 0.05 was considered the threshold of statistical significance.

## Results (Table 1)

### Neurogenic biopsy findings

Extensive grouping of normal sized type IIA fibers (Fig 1) was a common finding in the SMAJ and SBMA biopsies (in 58% and 40% of biopsies, respectively). These large non-atrophic IIA groups were 100% specific for the benign motor neuron disease group, as they were not encountered in any of the 12 c9ALS biopsies. Interestingly, these groups had already developed early in some SMAJ/SBMA patients with normal strength and short duration of disease, which is of importance regarding the time point of diagnostic evaluations. The presence of non-atrophic IIA groups declined over time, as 14/18 (78%) of SMAJ/SBMA displayed them when disease duration was 0–5 years as compared with 4/16 (25%) of biopsies performed in patients with disease courses of 6–24 years. Grouping of non-atrophic IIA fibers was not restricted to only certain anatomical muscles, as it was found in vastus lateralis, tibialis anterior and deltoid. In c9ALS, small group atrophy and atrophic groups containing mixed fiber types were common features. Fiber type grouping was usually only mild and focal in c9ALS.

Groups of highly atrophic rounded type IIA fibers were frequently observed in SMAJ/SBMA, but rarely in c9ALS patients.

### Other muscle pathology findings

Although all biopsies showed findings compatible with a primary neurogenic mechanism”secondary myopathic” changes were also frequent findings. Fatty replacement, fibrosis, internal nuclei and fiber splitting were commonly observed in all 3 groups, but when present, these findings were usually more abundant in SMAJ/SBMA. Hypertrophic fibers, defined as markedly hypertrophic (muscle fiber diameter >130 micrometers) or mildly hypertrophic (muscle fiber diameter 100–130 micrometers) occurred most commonly in SMAJ/SBMA but were also observed in some c9ALS biopsies.

Neonatal myosin heavy chain (MHCn) positive fibers were found in all biopsies, ranging between 1%-50% of the total number of fibers (median 12.5%) in c9ALS, 10%-50% (median 20%) in SBMA and 3%-50% (median 15%) in SMAJ. As expected in a neurogenic disorder, fetal myosin heavy chain (MHCd) positive fibers were much less frequent, with a median of 1% of fibers in all groups, ranging between 0%-5%, 0.5%-10% and 0%-20% of fibers in c9ALS, SBMA and SMAJ biopsies, respectively.

**Fig 2:**
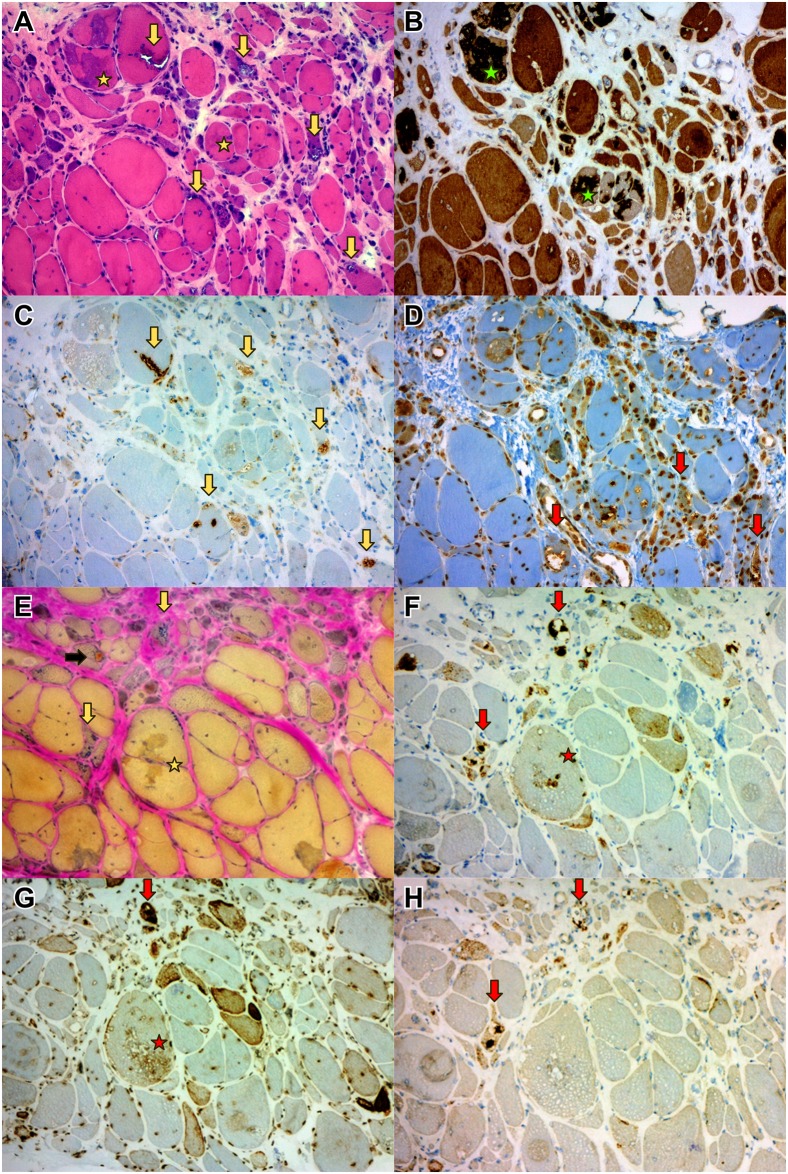
Secondary “myopathic” changes in *SMAJ*. 2: Unusually pronounced myofibrillar and autophagic pathology in a gastrocnemius medialis biopsy of a 67-year-old female with SMAJ. H&E and herovici stainings (A and E) showing endomysial fibrosis, fiber size variation, internal nuclei, as well as large myofibrillar aggregates (asterisk, A-B, E-G), several rimmed vacuolated fibers (vertical arrows) and one”cytoplasmic body” aggregate (black horizontal arrow). The myofibrillar aggregates are strongly myotilin immunoreactive (B) and show also some reactivity for p62 and SMI-31 (F and G). The rimmed vacuolar areas contain LC3 (C) and ubiquitin (D) positive material (with our antibody and protocol nuclei are also stained besides ubiquitinated cytoplasmic material). The rimmed vacuolated fibers are also strongly reactive for p62, SMI-31 and TDP-43 (F-H).

**Fig 3:**
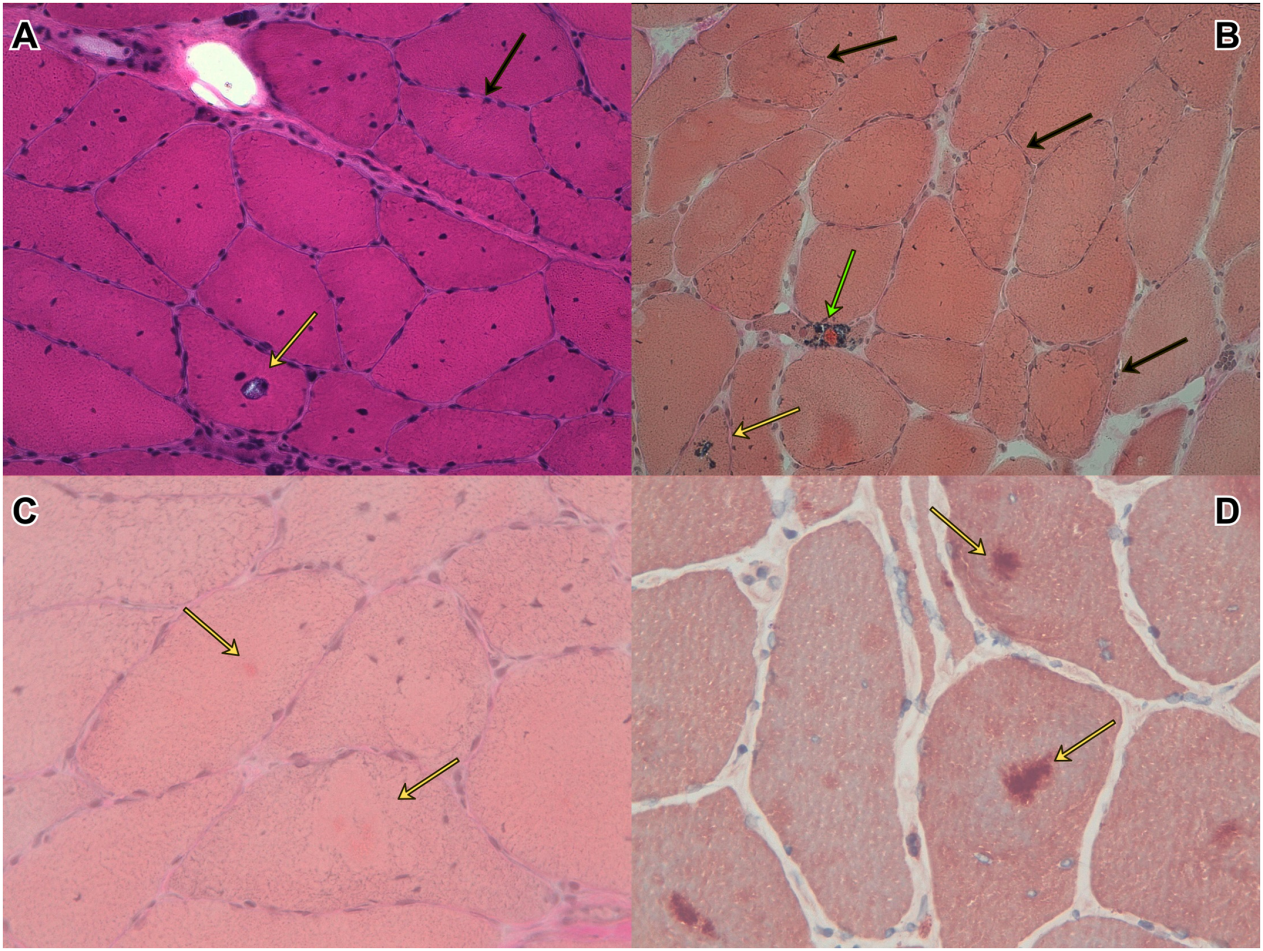
Secondary “myopathic” changes in *SBMA*. Muscle biopsy from a vastus lateralis biopsy of a 46-year-old male patient with SBMA showing internal nuclei, rimmed vacuoles (RV, yellow arrows, A-B), one RV and”cytoplasmic body” aggregate (green arrow), core-lobulated fibers with myofibrillar aggregates (black arrows in A-B, yellow arrows in C) that are strongly myotilin immunoreactive (yellow arrows, D).

Fibers with poorly defined moth-eaten areas of myofibrillar disorganization on oxidative enzyme stainings were a common biopsy finding in all disease groups. Occasional muscle samples in all disease groups contained fibers with core-like areas and lobulated fibers, but only focally. Target and/or targetoid fibers were relatively rare, and were not restricted to any disease group, but were more common in c9ALS samples than in SMAJ and SBMA. Rimmed vacuolated fibers were present in 7 SMAJ, 4 SBMA and 4 c9ALS patient muscle biopsies, although in only 2 SMAJ, 1 SBMA and 1 c9ALS patient biopsies were present in several fibers. 2 patients in the SMAJ group and 2 in the SBMA group showed prominent myofibrillar protein aggregate pathology on H&E and Gomöri trichrome -staining. In the SBMA group, myofibrillar aggregates and rimmed vacuolar pathology were most prominent in the patient with the longest disease duration (20 years) and in the one carrying the highest CAG-repeat number of 53 repeats (Fig 2). For 2 SMAJ and 1 SBMA patient samples with more prominent rimmed vacuolar pathology, we performed further immunohistological evaluations for Z-disc components (myotilin, desmin, alphaB-crystallin, BAG3), dystrophin carboxy terminal (DYS-2) and autophagy markers (LC3b, VCP, TDP-43, SMI-31, p62). The results are shown in Table 3 and Figs 2 and 3. The myofibrillar lesions were strongly myotilin and alphaB-crystallin immunopositive and ectopic dystrophin expression was usually present in the same fiber regions. In the rimmed vacuolated fibers protein aggregates were immunoreactive for markers of autophagic processing, including ubiquitin, p62, SMI-31, TDP-43, VCP and LC3b.

**Table 3:** Additional immunohistochemical studies in *SMAJ* and *SBMA* patients with rimmed vacuoles and/or myofibrillar pathology. ND = not defined, alphaBC = alphaB-crystallin, Dys-2 = dystrophin c-terminus, SMAJ = SMA Jokela type, SBMA = spinal and bulbar muscular atrophy, RV = rimmed vacuoles, CA = “cytoplasmic body” aggregates, VL = vastus lateralis, Gcmed = gastrocnemius medialis. -, normal or no immunoreactivity; +, immunoreactivity present/mild abnormality; ++ moderate immunoreactivity/abnormality; +++, high immunoreactivity/abnormality.

	SMAJ #1 (Soleus)	SMAJ #2(Gcmed)	SMAJ #3(VL)	SBMA #1(VL)
Appearanceon routinestains(H&E,Gomöri)	RV +++, secondarymyopathic/dystrophicfeatures	Myofibrillaraggregates++, CA ++,RV +++	RV +, mildmyofibrillaraggregates	RV ++,CA++,myofibrillar aggregates
Myotilin	+	+++	+	+
AlphaBC	+	++	+	+
Desmin	-	+	-	+
Dys-2	+	++	-	+
SMI-31	+++	+++	+	-
TDP-43	+++	+++	-	+
P62	+++	+++	+	+
Ubiquitin	++	++	+	+
LAMP-2	slightly increased	increased	normal	normal
VCP	+	++	-	-
FHL1	ND	-	ND	ND
LC3b	+++	+++	+	+

Minor, focal inflammatory infiltrates were observed in 4 SMAJ and one c9ALS biopsy and weak, patchy sarcolemmal staining with MHC class I antibodies was present in 5 SMAJ and 4 c9ALS biopsies. A few ragged red and COX-deficient fibers were an occasional finding in all 3 patient groups. The total number of ragged red fibers and COX-deficient fibers did not exceed 1% or 3% of the total number of fibers, respectively, in any of the muscle samples.

### *CHCHD10* immunohistochemistry and ultrastructural evaluation of SMAJ biopsies

**Fig 4:**
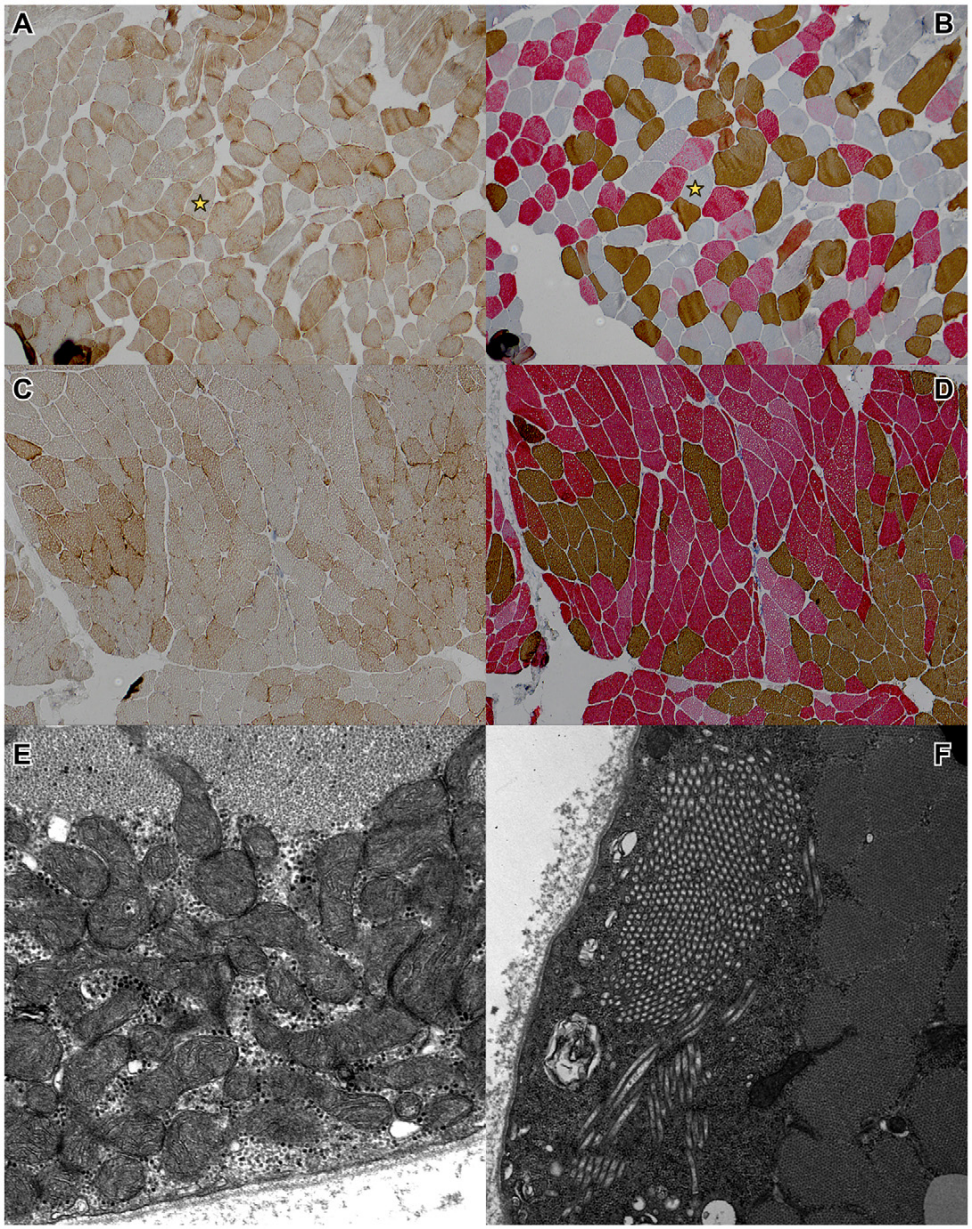
Lack of mitochondrial abnormalities in *SMAJ*. Immunohistochemical CHCHD10 staining in normal muscle sample (A) and muscle sample from SMAJ patient (C) showing no difference in expression and location of CHCHD10. The CHCHD10 staining is more prominent in type 1 fibers. The corresponding myosin double stainings are shown of the same muscles (B, D). Electron micrographs from deltoideus muscle of a 42-year-old SMAJ patient show morphologically normal mitochondria beneath the sarcolemma (E) and a subsarcolemmal tubular aggregate (F).

Because of the unexpected lack of mitochondrial muscle pathology in SMAJ in contrast to the findings previously reported with another CHCHD10 mutation [5], we further performed CHCHD10 immunohistochemistry and ultrastructural studies in 3 SMAJ patients to examine the mitochondria in more detail. In normal control muscle the mitochondrial CHCHD10 protein was more abundant in type I fibers, as expected. However, there was no difference in overall expression or localisation between normal and SMAJ patient muscle samples (Fig 4). For electron microscopy we selected patients with variable disease durations (less than 1 year in 2 and 7 years in 1), aged 42–67 years at the time of biopsy. The 67-year-old patient showed the most marked mitochondrial pathology of any SMAJ patient on light microscopic level, but displayed only 3% COX-deficient and 1% ragged red fibers. The other two patients showed only a few or no COX-deficient fibers. Ultrastructurally, the number and size of the mitochondria was in the normal range in all of the examined biopsies, and no abnormal mitochondrial aggregates were found. The morphology of cristae was within the normal range and no paracrystalline inclusions were identified. Only some of the mitochondria were degenerated corresponding to a nonspecific alteration in injured muscle cells.1 short duration SMAJ patient showed small subsarcolemmal tubular aggregates (Fig 4F), which were not evident on light microscopic evaluation, including NADH and Gomöri trichrome staining. The patient’s only symptom was widespread cramping of muscles.

## Discussion

The finding of extensive grouping with non-atrophic type IIA fibers was the major distinguishing feature in patients with the slow, less severe variants of motor neuron disease (SMAJ and SBMA), and was not observed in c9ALS patients. This is in line with previous observations that fiber type grouping without significant atrophy is associated with slowly progressive, well-compensated neurogenic processes [6]. However, the occurrence and significance of this important finding in adult-onset motor neuron diseases has not previously been appreciated.

Additionally we observed that the frequency of groups of non-atrophic IIA fibers declined over time in SBMA/SMAJ patients, as they were observed in 78% of patients with a disease duration 0–5 years, as opposed to only 25% of biopsies from patients with more protracted disease duration (6–24 years). The most obvious explanation for this would be that there is a better capacity for reinnervation at the early disease stages, and this process decreases with the continuous loss of motor neurons. By contrast, the presence of secondary myopathic changes seemed to increase with longer disease duration and caused diagnostic difficulties in some patients. The SMAJ/SBMA patients with most marked “myopathic” changes including prominent rimmed vacuolar pathology and high amounts (5–20%) of MHCd-positive fibers always had long disease durations (10–20 years) or, in one SBMA patient, an unusually large CAG-repeat. In addition, the second biopsy of the only c9ALS patient that was biopsied twice showed significantly more “myopathic” changes than the first specimen. In part these “myopathic” changes are likely to be adaptive or compensatory efforts by the myofibers, but in SBMA a primary myopathic component may also exist. [7]

Another mutation, c.176C>T p.S59L, in the CHCHD10 gene was reported with a different phenotype, frontotemporal dementia (FTD)/ALS with mitochondrial myopathy [5]. We have previously reported that SMAJ patients do not show major mitochondrial pathology in muscle at the light microscopic level, in contrast to the S59L-mutated *CHCHD10* patients with ALS/FTD [1]. The present study shows that the mitochondrial CHCHD10 protein is normally expressed on immunohistochemistry and confirms that the mitochondria of SMAJ patients were normal also when examined ultrastructurally.

Potential sources of bias in this work include the number biopsies in each group which is related to the availability of material, and that the re-evaluation was not blinded for the final diagnosis. Furthermore, only a proportion of patients with SMAJ and SBMA had undergone a muscle biopsy. However, the first routine diagnostic evaluation of biopsies nearly always predated molecular genetic diagnoses, and our non-biopsied SMAJ/SBMA patients had a very similar disease course as the biopsied ones selected for this study [8].

According to one study, atrophic groups consisting of mixed fiber types were more common in motor neuron disease biopsies (ALS and SBMA) than in polyneuropathies [9]. We found atrophic mixed fiber type groups to be frequent in c9ALS (75% of biopsies), while they were less common in SMAJ (33%) and SBMA (10%) biopsies. However, comparison with the previous work is not straightforward, because the authors did not report the results per patient but rather per high power fields examined [9].

The boundaries between myopathies, lower motor neuron disease and central nervous system disorders have recently become blurred, with the discovery of a *CHCHD10* mutation in mitochondrial myopathy associated with FTD/ALS (OMIM #615911), whereas the allelic disorder, SMAJ, causes a mild lower motor neuron disease and no cognitive decline. Furthermore, different mutations in *MATR3*-related disease may cause either ALS [10] or distal myopathy [11], and dementia/lower motor neuron syndrome can coexist with myopathies due to *VCP* mutations (OMIM #167320). Sporadic inclusion body myositis may also be confused with ALS clinically and on electromyography. All these syndromes differ significantly from classical ALS, and their proper identification is greatly facilitated by muscle histopathological investigations. Muscle biopsy may therefore be valuable in the diagnostic work-up of unclear motor neuron disorders, in order to provide patients with an accurate diagnosis and prognosis.
